# 601. Burn Units & Bubble Wands: Expanding Child Life Where It's Needed Most

**DOI:** 10.1093/jbcr/irag033.405

**Published:** 2026-04-06

**Authors:** Lindsey T Harris, Carey K Lamphier, Pamela Vanderberg, Kristen Glasgow-Petela, Laura S Johnson, Lauren B Nosanov, Emily Roach

**Affiliations:** Walter L Ingram Burn Center, Grady Memorial Hospital, Atlanta, GA; Grady Memorial Hospital, Atlanta, GA; Grady Memorial Hospital, Atlanta, GA; Morehouse School of Medicine, Atlanta, GA; Grady Memorial Hospital / Emory School of Medicine / Morehouse School of Medicine, Atlanta, GA; Emory University School of Medicine, Atlanta, GA; Grady Memorial Hospital, Atlanta, GA

## Abstract

**Introduction:**

Over 50 burn centers in the American Burn Association (ABA) directory operate within primarily adult healthcare systems while also caring for pediatric patients. The American Academy of Pediatrics recognizes Certified Child Life Specialists (CCLS) as essential healthcare providers in pediatric settings, yet their presence in adult hospitals remains inconsistent. Given the challenges associated with burn injuries in children, child life services are critical for providing developmentally appropriate education, coping support, and family-centered care. A quality improvement initiative was created to address a gap in services by building a sustainable child life program based in an ABA-verified burn center within the busiest adult Level 1 trauma centers in the country. This program aimed not only to meet the clinical psychosocial needs of pediatric patients in an adult-focused environment, but also to prove the value of these services beyond traditional pediatric settings.

**Methods:**

Our program began with limited, part-time child life coverage for the burn center with an increase to a single full-time position in 2015. Early efforts included staff education, return-to-school programs, burn camp coordination, and a burn center playroom. The program expanded to two full-time CCLSs in 2024, financed through the Burn Program and integrated within the burn center’s Process Improvement team. The Child Life team now provides 12-hour rotating shift coverage Monday through Friday. Services provided span the entire pediatric burn patient experience, from the emergency department to the burn intensive care unit (ICU), step-down unit, and outpatient clinic, and have also expanded to provide consult-based support to trauma, palliative, neurology, and neonatal ICU service lines.

**Results:**

In the first year of our current coverage model with two full-time CCLSs, the team documented over 1200 patient encounters: 767 in the burn center (inpatient and outpatient), 333 in trauma, 80 in neurology/palliative care, and 26 in the neonatal ICU. The most common categories of visits provided were procedural preparation and support (49.0%) and therapeutic interventions (19.6%).

**Conclusions:**

Our experience demonstrates that a sustainable child life program can thrive while based in an ABA-verified burn center located in a primarily adult-focused hospital. Next steps include expansion of services into non-burn pre-operative clinics, adult transition care, mother-baby, and deeper neonatal ICU engagement.

**Applicability of Research to Practice:**

In line with the Association of Child Life Professionals’ value proposition, the program has improved pediatric care coordination, increased efficiency of pediatric procedures, enhanced pediatric patient and family support, and supported programmatic goals such as ABA verification readiness.

**Funding for the study:**

N/A.

